# 

**Published:** 1885-01-20

**Authors:** 


### INDEX.

A.

Abdominal viscera, diseases of the.......... 94
Abortion.......... 182, 228
Abscess of the lung.......... 97
A. C. E. Mixture.......... 57
Aconitine.......... 247
Aërial railway and car.......... 447
Agnostic tendency of doctors.......... 37
Ague.......... 35, 154
Air; is it colorless?.......... 391
Albumen in urine.......... 103
Albuminuria.......... 150
Alcohol.......... 47
Alcoholic hyperœsthesia.......... 174
Alum, burnt.......... 262
Aloes; a new cure for cancer.......... 148
Amblyopia.......... 147
Amenorrhœa.......... 149
American Medical Association.......... 76, 87, 196, 237
American Rhinological Association.......... 356
Anaconda as a pet.......... 192
Anal fissure.......... 110
Angostura Bitters.......... 74, 433
Animal, a great.......... 152
Animal excentricities.......... 352
Animals, painless death of.......... 192
Anisic acid.......... 470
Anæmia.......... 113, 264, 344
Anasthetic agents.......... 149, 184, 388
Anasthetic, water as an.......... 467
Anodyne ointment.......... 354
Anteflexion of the uterus.......... 241
Antiperiodics.......... 73
Antipyretic formula.......... 230
Antipyrine.......... 189, 206
Antiseptics.......... 233, 418, 480
Antiseptics in Vienna.......... 127
Anus, fissured.......... 348
Apomorphine.......... 154, 336
Arnica, accidents produced by.......... 63
Arnicated glycerole of cantharides.......... 74
Arsenic to nursing women.......... 465
Arsenic treatment of cancer.......... 121
Arsenic in anæmia.......... 264
Association, a new National.......... 395
Aspirator in hydrothorax.......... 148
Asthma.......... 26, 33, 146, 273, 307, 355, 393
Atkinson, death of Dr.......... 36
Atlanta City Physicians.......... 435
Atlanta Society of Medicine.......... 477
Atrophic conditions.......... 264
Atropine in epilepsy.......... 313

B.

Bacillus of cholera.......... 19
Bacillus, the comma of.......... 96
Bacteria as therapeutic agents.......... 375
Balloon stearing.......... 1'1
Bandage, silicate of sodium.......... 28
Banking symstem, our National.......... 311
Barker, Dr. Fordyce.......... 76
Bee stings.......... 350
Bellinger, Dr.......... 396
Bengal cholera pill.......... 394
Black draught.......... 234
Bladder, blood clots in.......... 230
Bladder, irritable.......... 349
Bladder, over-distended.......... 386
Bleed? shall we.......... 18
Blood, intravascular injection of.......... 179
Boils.......... 75
Braithwaite, death of Dr.......... 396
Breast, bandage for the.......... 126
Bright's disease.......... 328, 394
Bromidia.......... 473
Bronchitis.......... 70
Bullard, Dr. W. L.......... 88, 286
Bulloch, death of Dr. W. G.......... 275
Burns, a specific for.......... 313, 354
Butter fifty years old.......... 32
Buttermilk in sick stomach.......... 259
Buzzing noise from quinine.......... 349

C.

Cæsarian section.......... 91
Caffein, hydrobomate of.......... 387
Caffeine valerianate.......... 387
Calcium sulphide.......... 230
Calcium chloride.......... 468
Calf-weaner.......... 434
Calomel in otorrhœa.......... 141
Calomel in pneumonia.......... 20
Campbell, death of Dr. Robert.......... 395
Camphor monohomide in spermatorrhœa.......... 110
Cancer.......... 28, 121, 156, 187, 235, 276
Cannabis indica.......... 69, 149
Cannibalism.......... 68
Carbolic acid.......... 220
Carbolic ointment compound.......... 274
Carbuncle.......... 266, 281, 313, 338
Carothers, Dr. John S.......... 248
Case, an interesting.......... 361
Castor oil.......... 330
Catarrh.......... 65, 94, 169, 376
Catheter, mode of introducing a.......... 308
Caudal development.......... 232
Cerium oxalate.......... 368
Cephalalgia.......... 473
Cerebral surgery, successful.......... 263
Chancre.......... 349
Channoid.......... 113
Chaulmoogra oil.......... 468
Cheese poison.......... 412
Cheynes stokes breathing.......... 303
Chicago Gynæcological Society.......... 256
Children, convulsions in.......... 201
Children, dysentery in.......... 373
Children, health of.......... 206
Children, skin diseases of.......... 330
Children, summer complaints of.......... 265
Chills.......... 33, 35, 314
Chimney, a paper.......... 432
Chimpanzee, an educated.......... 271
Chinese, health of the.......... 269
Chloroform water.......... 339
Cholagogue, digestive.......... 25
Cholera.......... 29, 235, 267, 342, 348, 353, 356, 394
Cholera bacillus.......... 19
Cholera infantum.......... 69, 291, 348, 385, 388
Cholera, prevention of.......... 475
Clavicle, fracture of.......... 228
Clingman, Hon. T. L.......... 10

Clinic, remarks made at a.......................................6
Clymer's Mixture for Asthma.........................273
Coca..............................................27, 190
Cocaine............19. 21, 28, 36, 59, 70, 88, 140, 144, 149, 173
209, 225, 274, 298 315, 346, 390, 459, 466, 467, 468, 470
Cocaine habit, dangers of.................................459
Coffee, extract..........................................273
Cold.............................................149, 211, 347
Colic.........................................................34
Congress, International Medical.................396, 397
Conia, hydrobomate of.....................................66
Constipation..................................................355
Convallaria majabis.......................................366
Convulsions ..............................................142, 200
Convulsions, uremic.........................................458
Copper, oleate of...........................................302
Cord, umbilical..............................................227
Corpulence.......................................................62
Corrosive sublimate........................................ 347
Corteylon, Dr. P. R.........................................206, 328
Coryza..........................................................193, 194
Cough..............................33, 34, 194, 214, 234, 274
Cough preparations.......................................194, 274
Counter-irritants..............................................153
Country, our......................................................431
Creasote water..................................................227
Creasote water as a local anasthetic.......................461
Croton oil, new vesicating..................................309
Croup..............................................................60
Crow, Dr. W. A..................................................125
Cunningham, death of Dr. F. D..........................395

D.

Dandruff...........................................................174
Darling, death of Professor......................................36
Debility, sexual.................................................434
Decapitated, experiments in...................................431
DaCosta, Professor............................................449
Delirium tremens ................................................ 33
Dental nerves, protection of...................................430
Dentition, application to the gums in.......................394
Diabetes ...........................................144, 167, 188
Diagnosis, a few points of......................................104
Diarrhœa.............................................33, 226, 314
Diet in headache.................................................. 229
Diphtheritic membrane, distribution of.......................93
Discrimination.....................................................236
Disease, germs of...................................................30
Disfranchising the Poor.........................................235
Disinfectant, a cheap and efficient...........................430
Dislocation.........................................................30, 150
Diuretic mixture.....................................................233
Dixon, Dr. Arch......................................................201
Doctor, an aged ......................................................315
Doctors, agnostic tendency of.....................................37
Doctors disagree ......................................................232
Doctors in the United States, number of......................357
Doctors in Atlanta ....................................................396
Doctors, some lay advice to.........................................467
Dosing, indiscriminate...................................................69
Dover's Powder and its modifications..........................460
Dover's powder syrup....................................................150
Dover's solution............................................................257
Drainage tubes...............................................................268
Dressings, surgical...........................................................287
Dropsy..............................................................................347
Durham, death of Dr. W. W............................................36
Dysentery.................................58, 113, 146, 233, 350, 393
Dysmenorrhœa...................................................... 99
Dyspepsia.......................................................345, 355
Dystocia from anomaly of fœtal skull.....................89

E.

Ear, foreign bodies in the.....................................419
Earache.............................................188, 209, 353
Ears, diseases of the................................................94
Eccentricities, animal.............................................352
Eclectic Journals......................................................357
Eczema..........................................................114, 234
Eczema, chronic squamous......................................468
Eczema, infantile.............................................453, 466
Electric lamp......................................................2[illegible]2
Electric light.....................................................72, 112
Electricity and Dust.................................................432
Electricity in disease.................................................74
Electricity in obstetrics.............................................. 146
Emissions, nocturnal.........................................315, 355
Enteralgia...................................................................248
Enuresis......................................................................384
Epilepsy ...............................................................66, 313
Epithelioma, arsenic treatment.......................................448
Ergot in dysentery..........................................................146
Erysipelas...........................................................34, 64, 105
Eserine.............................................................................109
Ether, anæsthesia by........................................................262
Ethics and Jurisprudence ................................................ 356
Ethyl, bromide of ............................................................250
Expectorant........................................................................34
Euthanasia.........................................................................438
Eye, to remove lime, etc., from the...................................465
Eyelids, eczema of.............................................................[illegible]14
Eyes, blue...........................................................................31

F.

Feet, bromidosis of the..........................................189
Feet, parœsthesia of the.........................................174
Ferran's inoculations.............................................429
Fever....................................................24, 153, 349
Fever, icteroid.........................................................403
Fever, intermittent....................................................349
Fever, malarial ..................................24, 263, 378, 457
Fever mixture..........................................................153
Few, death of Dr...............................................156, 276
Flame ...................................................................231
Flatulence...................................................................33
Flowers, Dr. G. E......................................................330
Fœtal Skull anomaly, dystoria from...............................89
Forceps, obstetric. .....................................................221
Forcite powder .........................................................391
Fossils; what they teach .............................................112
Fractures, Paris plaster in............................................372
Freckles........................................................................456
Freckle wash .................................................................434

G.

Gaillard, death of Dr.............................................76
Gall bladder, occlusion of..................................81, 155
Gangrene from quinine injection...............................109
Gas, industrial use of................................................ 27
Gaston, Dr. J. McF.................1, 48, 76, 81, 121, 161, 195
210, 235
Gaston's Operation...................................................37
Gastric ulcer..........................................................61
Geigley, Dr. J. S......................................................281
Gelseminum habit.....................................................424
Genitals, pruritus of the ...........................................274
Georgia Medical Association.................38, 115, 195, 197
Germ theory ...........................................................104
Germs, minuteness of...............................................392
Germs of disease.......................................................30
Gibbons, death of Dr. Henry........................................36
Ginger ale.................................................................193
Gleet.......................................................................434
Glycerine.....................................................229, 230
Glycerinum aluminis...................................................274
Goidtsnoven, Dr. E. van.....................................167, 321
Goitre..................................................29, 269, 341
Gokhru......................................................................469
Gonorrhœa........................144, 194, 270, 310, 418, 420
Gonorrhœa in female...................................................418
Graham, Dr. C. C.........................................................315
Grant, health of General ..............................................115
Gray, death of Dr. S. H..................................................395
Gynecology, cocaine in..................................................466

H.

Hair dye.........................................................434

Hair, falling.....................................................434

Hair tonic....................................................74, 223

Halo.................................................................232

Hæmoptysis.......................................................355

Hand, the..........................................................417

Hardon, Dr. Virgil O...........................................402

Harrell, death of Dr. J. D......................................36

Hay fever.............................................30, 147, 253

Head, cold in the.........................................347, 418

Headache.........76, 186, 229, 385
Headache, podophyllin and hydrastin in.........468
Health bill before Congress.........77
Hemorrhage, post partum.........222
Hemorrhage, uterine.........54
Hemorrhage, witch hazel in.........22
Hemorrhagic malarial fever.........263, 378
Hemorrhoids.........114, 393
Hemorrhoids, treatment of.........448
Hemostatic agent, a new.........469
Hepatic disorders.........176, 374
Hiccough, a new remedy for.........470
Hiccough, obstinate.........150
Hindoo Time.........312
Hip dislocations.........428
Hobbs, Dr. A. G.........209
Hopeine (alkaloid from hops).........456
Hope on, hope ever.........436
Horehound, compound syrup of.........74
Huchard's anti-tubercular pills.........394
Human being, caudal development in a.........232
Humerus dislocation.........39, 150
Humming of telegraph wires.........232
Humphries, Dr. B. Frank.........403
Hung, the pain of being.........311
Hydronaphthol.........426
Hydrastin and podophyllin.........468
Hydrastis ointment, compound.........73
Hydrophobia.........148
Hydrothorax.........148
Hygiene.........346
Hymeneal; Shaw—Gaston.........475
Hyoscine, hydrobomate of.........455
Hyosciamin, poisoning by.........416
Hypodermic syringe.........214, 420
Hypophosphite of lime in cancer.........187
Hysteria.........34

I.

Icteroid fever.........403, 442
Icterus.........167
Ileo-colitis.........433
Indigestion.........220
Infants, water for.........270
Influenza.........115
Ink stains.........193
Insane, testimony of the.........303
International Medical Congress.........155
Intestinal occlusion, death caused by.........307
Intra-uterine diseases.........141
Intus-susception.........2[illegible]9
Invention, a new Sweedish.........351
Iodine, medical uses of.........102, 307
Iodoform.........64, 188, 227
Iodoform in diabetes.........188
Iodoform in erysipelas.........64
Iodoform, antidote for.........227
Ipecac in indigestion.........181
Iridium.........111
Iris and evonymus.........383
Ink Indian.........392

J.

Jaborandi.........100, 150, 309, 417
Jaborandi in obstinate hiccough.........150
Jaborandi in pneumonia.........100
Jaborandi, overdose of.........309
Jaborandi, therapeutics of.........417
Jacaranda lamifoliata.........149
Jacobi, Dr.........235
Journal, Our.........442

K.

Kairin in the treatment of fever.........206
Katharion, Lyon's.........273
King, Dr. T. D.........52
Koch, cholera bacillus.........19, 70, 96
Kola.........106
Koller, Dr.........87

L.

Labor.........56, 60, 140, 250, 256, 306
Labor, bromide of ethyl in.........250
Labor, chloroform in.........140
Labor, functions of the membranes in.........256
Labor, muriate of cocaine in.........56
Labor, induction of premature.........306
Labor in a girl eleven years and nine months of age.........60
Lallerstedt, Dr. T. L.........126
Lancet, the.........77
Laparatomy.........
Laryngeal catarrh.........65
Laryngology.........70
Las Vegas, climate of.........57
Lawson, Dr. H. M.........156
Lawyers in the United States, number of.........357
Laxative sugar.........394
Laxative syrup.........393
Lead colic.........4[illegible]3
Light in front of the horse, put your.........472
Los Angeles, health of children in.........106
Louisiana State Medical Society.........115
Lung, abscess of the.........97
Lung, extirpation of the.........390
Lupus.........68
Lusk's Aperient Pills.........194
Lyon's Katharion.........273

M.

Magnesia, sulphate, in dropsy.........347
Malaria.........385
Malarial fever.........24, 263, 378, 457
Malarial hæmaturia.........305
Mangifera indica.........108
Match-splitting machine.........352
Medical Association of Texas.........470
Medical Press and Circular of London.........155
Medical prizes.........269
Medical societies.........115
Medlock, Dr. J. B.........448
Meningitis.........115, 161, 195, 210
Menorrhagia.........33, 428
Menthol.........467
Mercury, oleate of.........374
Mercury, volitile.........231
Metal, another new.........431
Meteoric dust.........112
Methyl, chloride of.........190
Michigan State Medical Society.........86
Microphotoscope.........151
Milk secretion.........230, 427
Milk secretion, deficient.........230
Mind-cure.........304
Ministers in the United States, number of.........357
Mississippi Valley Medical Society.........275
Mistletoe, a partarifacient.........419
Mixture, the A. C. E.........57
Monsel's Solution in diarrhœa.........226
Moon halo.........232
Morphine.........142, 348, 388
Morphine for quinine.........396
Morphine in uremic convulsions.........488
Mosquitoes, how to keep away.........430
Mouth, Nurse's sore.........154
Mumps, microbe of.........429
Murder? What is.........464
Muscular attachment, rupture of.........125
Myoma, a case of.........402

N.

Nævus, sodium ethylate in.........389
Naphthaline.........344
Nasal catarrh.........94
Nasal polypi.........61
Nausea and vomiting.........35
Negro exempt from hay fever.........253
Negro population.........272
Nephoritomy.........466
Nerves, uniting of different.........470
Nervous prostration.........75
Neuralgia.........114, 147
New Orleans Exposition.........356
Nipples, cracked.........467
Nipples, sore.........474
Nose, diseases of.........94

Nurse's sore mouth....154
Nursing women? Should arnica be given to....455

O.

Obstetrical Society of New York....89
Obstetrics, electricity in....146
Occlusion, intestinal....307
Oil spot....71
Ointment, compound carbolic....274
Oleomargarine....68
Oophoritis, double....93
Ophthalmia....233
Opium habit....147
Opium poisoning, a case of....254
Oranges for deficient milk secretion....130
Osmic acid....107
Os uteri, lacerations and fissures of....450
Otorrhœa, calomel in....141
Ovarian menorrhagia....428

P.

Paper in Tonkin....472
Paralysis....309, 350
Paralysis, agitans....350
Paris, plaster of, in fractures....372
Pasteur, Louis....17
Pepsin....189, 230
Peritoneum, tapping the....92
Perosmic acid....150
Petroleum in Russia....191
Pharyngo, nasal catarrh....94
Phenylhydraizine....148
Piles....216, 348, 350
Pill, how to take a....340
Piscidia....189
Placenta, how, when and where to deliver the..137
Platinum....232
Platinum crucibles....152
Plymouth epidemic, typhoid fever....295
Pneumonia....20, 100
Podophyllin and hydrastin....468
Polypi, nasal....61
Potassium permanganate in amenorrhœa....149
Potassium saltsylate....354
Powder, Forcite....391
Powell, Prof. Thomas S....157
Pox, small....395
Pregnancy, vomiting in....429, 470
Premium offer....475
Prize offered....115
Profession, to the....76
Professor and the Quack....45
Prohibition in Atlanta....437
Prolapsus ani....216
Proposition, a liberal....435
Prostatatomy....348
Pruritus....154, 167, 274
Psoriasis....377
Psoriasis, salicylic acid and castor oil in....467
Puerperal septicemia....26
Puget Sound, climatolgy of....351
Pulmonary consumption....449
Pun, an unintentional....145
Pyridin....307
Pyrophore....471

Q.

Quinia, bihydrobromate of....460
Quinine, hypodermic injections of, in malarial fevers....457

R.

Rabbit's eye....275
Railway and cars, ærial....448
Rainbow....232
Red drops....258
Remedies, proprietary....470
Renal affections....194
Reputation of a medical brother....361
Resorcine....67
Retina, atrophy of....27
Rheumatism....30, 75, 220, 354, 387
Rhinological Association, American....356
Rhus poisoning....355, 469
Richardson, Dr. Richard....54
Ringworm....62, 193, 314
Rod, divining....191
Rothelm, or German measles....414
Roy, Dr. G. G....241

S.

Sabonblene's tisane....194
Salt baths in fever....349
Salicylic acid and castor oil....467
Salicylate of soda....110, 186
Sassafras, poisoning by oil of....423
Scarf pin, electric light as a....112
Scarf pin, swallowing a....465
Scarlet fever....337
Sciatica....70, 107, 150
Scutellaria larerifolia....384
Searcy, death of Dr....356
Septicemia, puerperal....26
Sex of the child before birth....145
Shoemaker, Dr....235
Shoulder presentations....260
Silicate of sodium bandage....28
Silver, nitrate, in dysentery....58
Simaba Cedron in hydrophobia....148
Sims' memorial fund....469
Skin diseases in children....330
Sleeplessness, cocaine in....140
Slicer, A....352
Smith, Dr. T. C....50
Snake bite....76
Snakes....231
Snow-water impurities....72
Sodium salicylate....381
Sodium ethylate....389
Soleina....311
Soliloquy....435
Southern Medical College....76, 156, 395
Southern Medical Record....37, 116, 275, 357, 436
Specific, another....30
Spermatorrhœa....110
Spleen, enlarged....188
Sprain, elastic bandage for....25
Stanford, death of Dr. F. A....395
Stomach, wound of the....106
Stone in female bladder....135
Strabismus....286
Strychnine, antidote to....188
Stuttering....423
Subscribers, new....473
Sugar in ohthisis and dysentery....350
Sugar in urine....103
Surfaces, inflamed....354
Surgeon-General, report of....476
Surgery, cerebral....463
Surgery, cocaine in minor....144
Surgical dressings....287
Surgical instrument, a new....352
Swamp button-bush in rhus poisoning....469
Sweating in ohthisi....113
Syncope from the use of cocaine....173
Syphilis, hereditary....24, 433
Syringe, hypodermic....214
Syrup, laxative....393

T.

Tænicide, glycerine as a....310
Tampon, use and abuse of....182
Tamponing the nasal fossæ....346
Tannin acid....348
Tape-worm....M.... 21, 27, 420
Telegraph wires, humming of the....232
Telescope....152
Temperature of the World....31
Terpine....30
Tetanus, traumatic....261
Tetter ointment....73
Texas State Medical Association....36, 115
Thallin....108
Thirst in febrile states....229
Thoracic viscera, diseases of the....94
Throat, sore....34, 94, 173

Tinea capitis....302
Titanium, or cimicifuga....22
Toads, a new use of....351
Tobacco....10, 190
Toe nails....52, 228, 348
Tomatoes, dyed....432
Tongue, dryness of....229
Tonkin, paper in....472
Tonsilitis....428
Toothache drops....394
Tooth extraction, painless....294
Transactions of Georgia Medical Association...195
Treatment, notes of Dr. DaCosta on....449
Tuberculosis and vaccination....226
Tumors, turpentine in malignant....469
Turning, the operation of....429
Turpentine, medical and surgical uses of....41
Turpentine plugs....346
Twins born four days apart....150
Tympanites....92
Tpyho-malarial fever....130
Typhoid epidemy....275, 295
Typhoid fever....225, 344, 421
Tyrotoxicon, cheese poison....412

U.

Ulcer, gastric....61, 143
Umbilical cord....227
Unna's bougie treatment....420
Urethral discharges....430
Urethan....462
Uremic convulsions....458
Urinary and generative organs....408
Urine, albumen and sugar in....103
Urine, retention of....330
Urine, suppression of....466
Uterine diseases....141
Uterus, action of warm water upon gravid....310
Uterus, anteflexion of the....241
Uterus, salicylate of soda upon the....110
Uvula, elongated....113

V.

Vaccination....224, 226, 270
Vaccine in yellow fever....61
Variola and vaccinia coincident....389
Variola incubation, vacciuation during the....224
Varnish, to remove....272
Veratrum as a diaphoretic....429
Verdery, Dr. C. S....361
Vesical affections....194
Vinegar in bowel complaints....15
Vinegar in post mortem hemorrhage....222
Vinegar, uses of....462
Viscera, protrusion of the....106
Vomiting and nausea....35, 453, 368
Vomiting, cocaine in obstinate....468
Vomiting of pregnancy....429, 470
Vulvæ pruritus....154

W.

Wakefulness....114
Warts, venereal....347
Washington, Mount....192
Water, a local anesthetic....469
Water for infants....270
Waugh, Dr. Thomas....395
West Texas Medical Association....470
Whooping cough....34, 69, 273, 470
Winckler, Professor....70
Winds and the weather....71
Witch hazel in hemorrhage....22
Wizzard oil....234
Word, Dr. R. C....6
World, temperature of the....31
World's Exposition....155
Wounds, treatment of....50
Wound dressing....133
Wright, Dr. T. S....47

Y.

Yellow fever, vaccine in....61, 270, 436

Z.

Zinc, phosphate of....99

### EDITORIALS AND MISCELLANEOUS.

### EDITORIAL NOTICES.

CAUTION.—we feel it to be a duty to our readers to sav that we have good reasons for believing that *The Franklin Mills Company, of Chicago*, is a swindling concern. Beware, therefore, of the concern, and of the flour which they are vending, as a corrupt tree is not likely to bring forth good fruit.

DEATH OF DR. DURHAM.—Dr. W. W. Durham, long a citizen and well-esteemed physician of Decatur, Georgia, died at his residence on the 27th ultimo, of diabetis—aged 63 years.

DEATH OF DR. J. D. HARRELL —We learn in a letter from his beloved wife that Dr. . D. Harrell, of Melvin, Ala., is no more. She writes that " he was a man of many noble traits of character," and that "his death was a public calamity, having been the beloved physician of the communitv for many years—a man of good works and a zealous Christian.

DEATH of PROFESSOR DARLING.—Prof. William Darling, the great Anatomist, of the New York University, died on Christmas morning, 1884.

DEATH OF DR. ATKINSON.—Dr. Atkinson, lecturer on Surgery and Genital Diseases in Long Island Hospital, died recently in Brooklyn, N. Y. He died a martyr to the profession, having contracted malignant syphilis from operating on a patient affected with that disease.

DR. HENRY GIBONS, of San Francisco, Cal., formerly editor of the Pacific Medical Journal, and a practitioner of eminence in the West, died recently at his home in San Francisco—aged 76 years.

THE Texas State Medical Association meets at Houston, on the third Tuesday of April, 1885.

THE Michigan State Medical Society meets at Port Huron, June 15, 1885.

HYDROCHLORATE OF COCAINE.—The discovery of this agent as a local anæsthetic in eve cases, was made by Dr. Koller, of Vienna, and reported to the congress of Ophthmalogy of Heidelburg. It was previously known, however, that the chewing of the leaf of the coca produced insensibility, and Shrotter, of Vienna, and Chauval and Panas, of Paris, had used it upon the larynx, throat and tongue.

COCAINE.—The new local anæsthetic, Cocaine, is at present exceedingly expensive. The last importation is said to have cost $249 per ounce.

SHARPE & DOHME.—See the advertisement of that splendid Drug House, of Baltimore, on second page of cover

PARVOULES.—Among the improvements and conveniences of modern pharmacy must be classed the *Parvoules* of that excellent and reliable Drug House of William R. Warner & Co., of Philadelphia. They have proven especially acceptable to the busy practitioner, in that he is enabled to grade the dose to any desirable quantity suited to any age. Being exceedingly small, and sugar coated, children will usually take them without trouble ; and, in the case of nervous and delicate females, and all that class of fastidious persons, who are so apt to take up with homœpathic methods, the Parvoules meet with ready acceptance. It is evident, from the certainty and efficiency of their action, that the Parvoules are prepared with great care, and of the very best materials.

### *RECEIPTED.*

1884.—Dr. I. B. Rutland; W. S. McFall; P. P. Terry; I. B. Lee; J. B. Foster; W. S. Johnson.

1885.—D. B. Hamilton; W. B. King; F. S. Gipson; T. M. Palmer; S. M. Hogan; W. S. Glass; R. L. Hinton; J. A. Pipkin; Z. J. Scott; J. E. Frippe; R. A. Shimpoch; R. B. Shelby; T. P. Oliver; J. W. Baker; W. C. Lamb; I. A. Gordon, '83; J. N. Wadsworth, '83; J. W. Talley, '83; I. H. McCaleb, '83; E. E. Scott; L. N. Miles.

